# Clinical significance of *PD-L1* and *PD-L2* copy number gains in non-small-cell lung cancer

**DOI:** 10.18632/oncotarget.8528

**Published:** 2016-04-01

**Authors:** Yusuke Inoue, Katsuhiro Yoshimura, Kazutaka Mori, Nobuya Kurabe, Tomoaki Kahyo, Hiroki Mori, Akikazu Kawase, Masayuki Tanahashi, Hiroshi Ogawa, Naoki Inui, Kazuhito Funai, Kazuya Shinmura, Hiroshi Niwa, Takafumi Suda, Haruhiko Sugimura

**Affiliations:** ^1^ Department of Tumor Pathology, Hamamatsu University School of Medicine, Hamamatsu, Shizuoka, Japan; ^2^ Second Division, Department of Internal Medicine, Hamamatsu University School of Medicine, Hamamatsu, Shizuoka, Japan; ^3^ Department of Pathology, Hamamatsu Medical Center, Hamamatsu, Shizuoka, Japan; ^4^ First Department of Surgery, Hamamatsu University School of Medicine, Hamamatsu, Shizuoka, Japan; ^5^ Division of Thoracic Surgery, Respiratory Disease Center, Seirei Mikatahara General Hospital, Hamamatsu, Shizuoka, Japan; ^6^ Department of Pathology, Seirei Mikatahara General Hospital, Hamamatsu, Shizuoka, Japan; ^7^ Department of Clinical Pharmacology and Therapeutics, Hamamatsu University School of Medicine, Hamamatsu, Shizuoka, Japan

**Keywords:** PD-L1, PD-L2, amplification, copy number, non-small-cell lung cancer

## Abstract

New reliable biomarkers are needed to predict the response to immune checkpoint inhibitors against programmed death-1 (PD-1) and its ligand (PD-L1), because PD-L1 expression on tumor cells has limited power for selecting patients who may benefit from such therapy. Here we investigated the significance of *PD-L1* and *PD-L2* gene copy number gains using fluorescence *in situ* hybridization as well as PD-L1 and PD-L2 expression in 654 patients with resected non-small-cell lung cancer. The prevalence of *PD-L1* amplification and polysomy was 3.1% and 13.2%, respectively. The *PD-L1* gene copy number status was in agreement with both the *PD-L2* and Janus kinase 2 gene copy number statuses. PD-L1 and PD-L2 expression was observed in 30.7% and 13.1%, respectively. Both PD-L1 copy number gains and expression were associated with smoking-related tumors. Tumor cells with *PD-L1* genomic gains exhibited significantly higher levels of PD-L1 expression than those without, but *PD-L2* copy number gains were not related to PD-L2 augmentation. *PD-L1* gene amplification and polysomy were independently associated with PD-L1 expression, with high immune infiltrates and EGFR expression in a multivariate logistic regression model. Comparative analysis between primary tumors and synchronous regional lymph node metastases revealed that the *PD-L1* gene copy number alterations were highly consistent and reproducible compared with the PD-L1 expression. Both *PD-L1* amplification and level of protein expression were predictors of poor survival using Cox univariate analyses. Therefore, we conclude that an increase in *PD-L1* gene copy number can be a feasible alternative biomarker for predicting response to anti-PD-1/PD-L1 therapy.

## INTRODUCTION

Treatment strategies for non-small-cell lung cancer (NSCLC) have three main components: surgery, radiotherapy, and chemotherapy. Of these, significant advances in chemotherapy have been made over the past decade, including the selection of new agents based on histology. Molecular targeted therapies for specific genomic alterations, such as epidermal growth factor receptor (*EGFR*) mutations and anaplastic lymphoma kinase (*ALK*) gene rearrangements, are among the greatest innovations in the field of personalized cancer therapy. However, the outcomes of NSCLC patients have not yet been improved satisfactorily. Lung cancer, of which NSCLC comprises almost 85%, is still a leading cause of cancer-related death worldwide [1]. Recently, cancer-targeted immunotherapies, such as immune checkpoint inhibitors, have evolved as a fourth treatment strategy. The latest clinical trials [2, 3] have shown that nivolumab, an anti-programmed death-1 (PD-1) fully humanized monoclonal antibody, has yielded unprecedented benefits in patients with NSCLC.

PD-1, also known as CD279 and a member of the CD28 family, is a co-inhibitory receptor that plays a crucial role in immune escape during tumor progression [4]. The PD-1 ligands such as PD-L1 (CD274) and PD-L2 (CD273) are B7 family members and are known to be overexpressed on the surface of tumor cells where they block cytotoxic T cells [4–6]. The interaction of PD-L1/L2 and PD-1 on activated T cells leads to the exhaustion of T cells via the inhibition of T-cell receptor signaling and co-stimulatory signals [7–10]. Blockade of the immunosuppressive PD-1/PD-L1 pathway to enhance T-cell responses has successfully shown significant antitumor activities in various cancers including NSCLC [2, 3, 11]. The expression level of PD-L1 on tumor cells has been suggested to be a positive biomarker predicting the efficacy of anti-PD-1 or -PD-L1 therapy [3, 12–14]. Two major mechanisms that explain the expression of immune-checkpoint ligands on tumor cells are known: adaptive immune resistance and innate immune resistance [15]. In adaptive immune resistance, PD-L1 expression on the tumor cell surface is induced by inflammatory signals, such as interferon-γ secreted by activated T cells [4, 16]. In innate immune resistance, oncogenic gene alterations in NSCLC, such as *EGFR* mutations [17, 18], *ALK* rearrangements [19], and *PTEN* loss followed by the activation of the PI3K–Akt pathway [20], induce PD-L1 expression leading to the inhibition of tumor cell destruction by immune cells. Other mechanisms of innate immune resistance are also likely to exist; however, the overall scheme controlling PD-L1 expression has never been addressed. Furthermore, little information is available concerning the mechanisms of PD-L2 expression in the innate state.

Copy number gains may be responsible for increased expression levels of genes located at the gained locus in the genome. In primary mediastinal large B-cell lymphoma [21], Hodgkin's lymphoma [21, 22], gastric cancer [23], and triple-negative breast cancer [24], the amplification of chromosome 9p24.1 containing the *PD-L1* and *PD-L2* genes as well as Janus kinase 2 (*JAK2*) is known; this suggests that the activation of JAK-STAT signaling may be partially involved in subsequent changes experienced by cells possessing this focal amplification. In contrast, the prevalence and significance of copy number gains of the specific *PD-L1* and *PD-L2* loci in NSCLC have yet to be clarified.

Here we investigated whether copy number gains of the *PD-L1* and *PD-L2* genes, as identified using fluorescence *in situ* hybridization (FISH), were correlated with the upregulation of the corresponding proteins and with patients’ survival outcomes using a large cohort comprising 654 resected patients with NSCLC. In addition, we performed comparative analysis of the gene copy number and protein expression of PD-L1 using specimens of metastatic regional lymph nodes and matched primary tumors, which were obtained from identical surgical resection, to assess consistency and reproducibility of *PD-L1* gene copy numbers and PD-L1 protein expression.

## RESULTS

### Status of PD-L1 expression and *PD-L1* gene copy number alterations

A total of 654 surgically treated patients with NSCLC were included. The tumors were histologically classified as adenocarcinoma in 430 (65.7%) cases, as squamous cell carcinoma in 179 (27.4%) cases, and as other histologies (adenosquamous carcinoma, *N* = 19; large cell neuroendocrine carcinoma, *N* = 11; pleomorphic carcinoma, *N* = 7, large cell carcinoma, *N* = 5; giant cell carcinoma, *N* = 2; and carcinosarcoma, *N* = 1) in 45 (6.9%) cases. PD-L1 was overexpressed in tumor cells in 201 (30.7%) cases. FISH analyses for *PD-L1* were successful in 636 specimens. The patient characteristics according to PD-L1 expression and *PD-L1* gene copy number status are shown in Table 1. Among patients with PD-L1-positive tumors, the proportions of male sex, smoking history, squamous histology, advanced nodal and disease stages, high immune infiltrates, high EGFR expression, high phospho-EGFR (p-EGFR) expression, and *EGFR* wild type were significantly higher than among those with PD-L1-negative tumors. Regarding the *PD-L1* copy number status, the numbers of cases were 20 (3.1%) for amplification, 84 (13.2%) for polysomy, and 532 (83.7%) for disomy. Polysomy was subclassified into high polysomy in 43 (6.8%) patients and low polysomy in 41 (6.4%) patients. Borderline polysomy was observed in 19 (3.0%) specimens. Among the *PD-L1*-amplified tumors, the mean value of the *PD-L1* signals ranged from 4.5 to 11.9 (median, 5.9), and the *PD-L1*/centromere enumeration probe (CEP) 9 ratios ranged from 2.1 to 4.9 (median, 2.6). Among tumors with *PD-L1* polysomy, the average *PD-L1* signal ranged from 3.0 to 8.6 (median, 4.0). Similar to cases with PD-L1 expression, *PD-L1* amplification and polysomy were associated with characteristics related to smoking. Interestingly, *PD-L1* amplification was not observed among tumors with either mutant EGFR expression or ALK expression.

**Table 1:** Clinicopathological characteristics of patients with non-small-cell lung cancer related to PD-L1 expression and *PD-L1* copy number status

	PD-L1 expression				*PD-L1* copy number status			
Characteristic	Total (*N* = 654) *N* (%)	Positive (*N* = 201) *N* (%)	Negative (*N* = 453) *N* (%)	*P* value	Amplification (*N* = 20) *N* (%)	Polysomy (*N* = 84) *N* (%)	Disomy (*N* = 532) *N* (%)	*P* value
Age (years)								
Median (range)	68 (23–88)	69 (33–85)	68 (23–88)	0.18	67 (44–84)	69 (33–85)	68 (23–88)	0.56
Sex								
Male	445 (68.0)	159 (79.1)	286 (63.1)	< 0.0001	16 (80.0)	70 (83.3)	349 (65.6)	0.0021
Female	209 (32.0)	42 (20.9)	167 (36.9)		4 (20.0)	14 (16.7)	183 (34.4)	
Smoking status								
Never	197 (30.1)	34 (16.9)	163 (36.0)	< 0.0001	2 (10.0)	11 (13.1)	177 (33.3)	< 0.0001
Ever	444 (67.9)	163 (81.1)	281 (62.0)		17 (85.0)	72 (85.7)	345 (64.8)	
Unknown	13 (2.0)	4 (2.0)	9 (2.0)		1 (5.0)	1 (1.2)	10 (1.9)	
Histology								
Adenocarcinoma	430 (65.7)	97 (48.3)	333 (73.5)	< 0.0001	5 (25.0)	45 (53.6)	369 (69.4)	< 0.0001
Squamous cell carcinoma	179 (27.4)	85 (42.3)	94 (20.8)		11 (55.0)	30 (35.7)	134 (25.2)	
Others	45 (6.9)	19 (9.4)	26 (5.7)		4 (20.0)	9 (10.7)	29 (5.4)	
p-T								
1	269 (41.1)	74 (36.8)	195 (43.0)	0.36	3 (15.0)	25 (29.8)	237 (44.5)	0.0011
2	283 (43.3)	92 (45.8)	191 (42.2)		10 (50.0)	43 (51.2)	223 (41.9)	
3	64 (9.8)	24 (11.9)	40 (8.8)		2 (10.0)	12 (14.3)	44 (8.3)	
4	38 (5.8)	11 (5.5)	27 (6.0)		5 (25.0)	4 (4.7)	28 (5.3)	
p-N								
0	481 (73.5)	131 (65.2)	350 (77.3)	0.012	7 (35.0)	58 (69.0)	405 (76.1)	< 0.001
1	77 (11.8)	31 (15.4)	46 (10.1)		9 (45.0)	11 (13.1)	57 (10.7)	
2	89 (13.6)	36 (17.9)	53 (11.7)		3 (15.0)	14 (16.7)	66 (12.4)	
3	7 (1.1)	3 (1.5)	4 (0.9)		1 (5.0)	1 (1.2)	4 (0.8)	
Pathological stage								
I	416 (63.6)	112 (55.7)	304 (67.1)	0.014	5 (25.0)	51 (60.7)	351 (66.0)	0.0023
II	113 (17.3)	39 (19.4)	74 (16.3)		7 (35.0)	13 (15.5)	91 (17.1)	
III	125 (19.1)	50 (24.9)	75 (16.6)		8 (40.0)	20 (23.8)	90 (16.9)	
Adjuvant chemotherapy								
Yes	266 (40.7)	85 (42.3)	181 (40.0)	0.61	10 (50.0)	37 (44.0)	210 (39.5)	0.49
No	388 (59.3)	116 (57.7)	272 (60.0)		10 (50.0)	47 (56.0)	322 (60.5)	
Intensity of immune infiltrates								
High	73 (11.2)	38 (18.9)	35 (7.7)	< 0.0001	4 (20.0)	7 (8.3)	61 (11.5)	0.32
Low	581 (88.8)	163 (81.1)	418 (92.3)		16 (80.0)	77 (91.7)	471 (88.5)	
EGFR intensity								
High	338 (51.7)	138 (68.7)	200 (44.2)	< 0.0001	14 (70.0)	52 (61.9)	267 (50.2)	0.037
Low	316 (48.3)	63 (31.3)	253 (55.8)		6 (30.0)	32 (38.1)	265 (49.8)	
p-EGFR intensity								
High	142 (21.7)	58 (28.9)	84 (18.5)	0.0040	4 (20.0)	27 (32.1)	111 (20.9)	0.075
Low	512 (78.3)	143 (71.1)	369 (81.5)		16 (80.0)	57 (67.9)	421 (79.1)	
p-Akt intensity								
High	27 (4.1)	9 (4.5)	18 (4.0)	0.83	1 (5.0)	3 (3.6)	23 (4.3)	0.82
Low	627 (95.9)	192 (95.5)	435 (96.0)		19 (95.0)	81 (96.4)	509 (95.7)	
Mutant EGFR expression								
Positive	132 (20.2)	25 (12.4)	107 (23.6)	0.0010	0	12 (14.3)	114 (21.4)	0.013
Negative	522 (79.8)	176 (87.6)	346 (76.4)		20 (100)	72 (85.7)	418 (78.6)	
ALK expression								
Positive	10 (1.5)	5 (2.5)	5 (1.1)	0.19	0	1 (1.2)	9 (1.7)	1.0
Negative	644 (98.5)	196 (97.5)	448 (98.9)		20 (100)	83 (98.8)	523 (98.3)	

Abbreviations: EGFR, epidermal growth factor receptor; p-EGFR, phospho-EGFR; p-Akt, phospho-Akt; ALK, anaplastic lymphoma kinase.

*P* values were obtained using the Mann–Whitney *U* test, the Kruskal–Wallis test, and the Fisher's exact test.

Fluorescence *in situ* hybridization analyses were not successful in 18 patients because of insufficient signaling intensity or loss of cores.

### Status of PD-L2 expression and *PD-L2* gene copy number alterations

**Figure 1:**
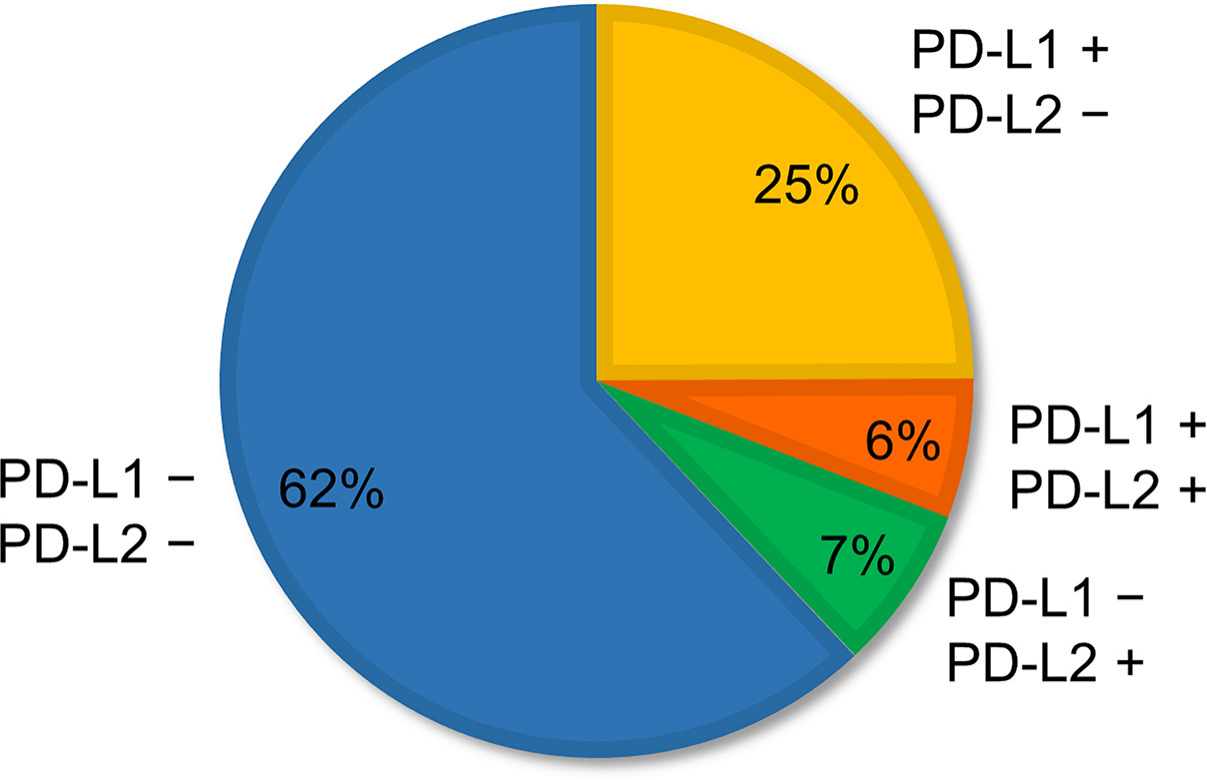
Proportion of patterns of PD-L1 and PD-L2 expression observed in non-small-cell lung cancer Of 650 specimens, 25% (***N*** = 162) were PD-L1 positive but PD-L2 negative; 6% (***N*** = 39) were both PD-L1 and PD-L2 positive; 7% (***N*** = 46) were PD-L1 negative but PD-L2 positive; and 62% (***N*** = 403) were both PD-L1 and PD-L2 negative.

The clinicopathological characteristics according to PD-L2 expression and gene copy number status are given in Supplementary Table S1. FISH analyses for *PD-L2* were successful in 635 specimens. Because of core loss, PD-L2 protein expression was not evaluated in four patients. In comparison with PD-L1, PD-L2 expression was observed less frequently (85 of 650, 13.1%). PD-L2 expression was associated with squamous histology, high immune infiltrates, high EGFR expression, and high p-EGFR expression. *PD-L2* amplification and polysomy were observed in 11 (1.7%) and 77 (12.1%) patients, respectively. The mean value of the *PD-L2* signals among the tumors with *PD-L2* amplification ranged from 4.0 to 9.9 (median, 5.7), and that of the *PD-L2*/CEP9 ratios ranged from 2.0 to 3.9 (median, 2.3). The average *PD-L2* signal among tumors with *PD-L2* polysomy ranged from 3.0 to 7.8 (median, 3.9). FISH signals sufficient for the interpretation of both the *PD-L1* and the *PD-L2* probe sets were observed in 628 tumors. The distribution of copy number alterations of the *PD-L1* and *PD-L2* genes among those tumors is shown in Supplementary Table S2. The weighted kappa coefficient for agreement between copy number alterations of *PD-L1* and *PD-L2*, which were subclassified into five groups, was 0.91 (95% confidence interval (CI), 0.87–0.94), indicating excellent concordance. Thus, the clinicopathological characteristics of patients with *PD-L2* copy number gains were almost the same as those with *PD-L1* copy number increase (Supplementary Table S1). In contrast, more than half of the PD-L2-positive tumors (46 of 85, 54.1%) were PD-L1 negative (Figure 1).

### Association of copy numbers between the *PD-L1* and *JAK2* genes

Of 636 specimens for which *JAK2*-FISH results were successfully obtained, *JAK2* was amplified in 16 (2.5%) specimens, 77 (12.1%) showed polysomy (high polysomy, *N* = 34 (5.3%); low polysomy, *N* = 43 (6.8%)), and 543 (85.4%) showed disomy (borderline polysomy, *N* = 37 (5.8%)). The mean value of the *JAK2* signals among the tumors with amplification ranged from 4.6 to 12.5 (median, 6.0), and the *JAK2*/CEP9 ratios ranged from 2.0 to 4.4 (median, 2.4). The average *JAK2* signal among tumors with polysomy ranged from 3.0 to 7.5 (median, 3.8). FISH signals of both the *PD-L1* and the *JAK2* probe sets were successfully interpreted in 625 specimens, and their distribution status is shown in Supplementary Table S3 (weighted kappa = 0.87; 95% CI, 0.83–0.91), where they exhibit excellent concordance. Next, we investigated whether an increase in *JAK2* copy number activated the JAK2/STAT3 pathway by evaluating the phosphorylation status of STAT3 as a surrogate for signaling activity in 634 specimens. However, no association was found among the copy number status of *JAK2* and the positivity of phospho-STAT3 (p-STAT3) expression (p-STAT3 was positive in two of 16 for tumors with *JAK2* amplification, in 21 of 77 for tumors with *JAK2* polysomy, and in 173 of 541 for tumors with *JAK2* disomy; Fisher's exact test, *P* = 0.20).

### Factors affecting PD-L1 expression

**Figure 2:**
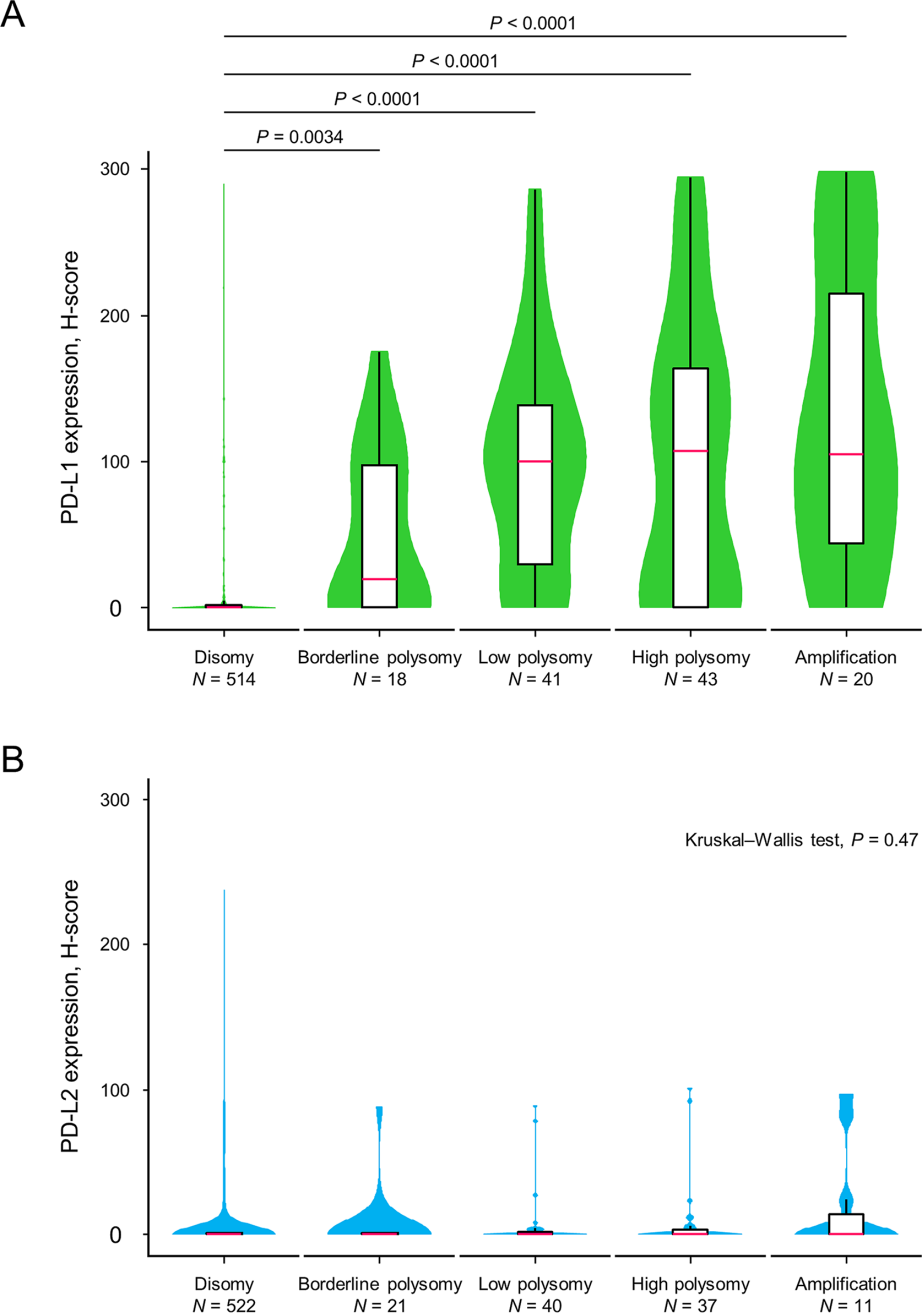
Violin plots with box plots showing PD-L1 and PD-L2 protein expression levels in relation to copy number alterations of the corresponding genes The green-colored shapes represent a kernel density plot of the distribution of the PD-L1 protein expression levels (**A**). The blue-colored shapes give a kernel density plot of the distribution of the PD-L2 protein expression levels (**B**). The box plot shows the median (pink line) and interquartile range (top and bottom borders of the box). The whiskers above and below the box represent 1.5 × the interquartile range. The Kruskal–Wallis test was used to examine differences in the PD-L1 and PD-L2 expression levels.

The PD-L1 protein expression levels according to the copy number status of *PD-L1* are shown in Figure 2A. Many tumors with *PD-L1* disomy had no or low levels of PD-L1 expression, with a median H-score value of 0 (interquartile range (IQR), 0–2). The expression levels of PD-L1 among tumors with *PD-L1* borderline polysomy (median, 19 (IQR, 0–98)), low polysomy (median, 100 (IQR, 30–138)), high polysomy (median, 107 (IQR, 0–164)), and amplification (median, 105 (IQR, 44–215)) were significantly greater than those with *PD-L1* disomy. In a multivariate logistic regression analysis (Table 2), *PD-L1* amplification (odds ratio (OR), 14.20; 95% CI, 3.90–52.00) and *PD-L1* polysomy (OR, 10.60; 95% CI, 5.96–18.80) were independently associated with PD-L1 expression. High immune infiltrates (OR, 3.50; 95% CI, 1.95–6.27) and EGFR expression (OR, 2.14; 95% CI, 1.37–3.36) were also independently associated with PD-L1 expression, and ALK expression tended to be associated with PD-L1 expression (OR, 3.74; 95% CI, 0.95–14.70). Representative cases with no PD-L1 expression and disomy of both *PD-L1* and *PD-L2*, PD-L1 overexpression and polysomy of both *PD-L1* and *PD-L2*, and PD-L1 overexpression and amplification of both *PD-L1* and *PD-L2* are presented in Supplementary Figure S1. Distinct from PD-L1, PD-L2 expression levels did not increase according to *PD-L2* copy number gains (Figure 2B).

**Table 2:** Results of univariate and multivariate logistic regression analyses of clinicopathological and molecular factors predicting PD-L1 positivity

Variable	Per unit for OR	Unadjusted OR	95% CI	*P* value	Adjusted OR	95% CI	*P* value
Sex	Male/female	2.21	1.50–3.26	< 0.0001	1.13	0.59–2.16	0.72
Smoking status	Ever/never	2.78	1.83–4.22	< 0.0001	1.15	0.57–2.32	0.70
Histology	Squamous cell carcinoma/adenocarcinoma	3.10	2.14–4.50	< 0.0001	1.54	0.92–2.59	0.098
	Others/adenocarcinoma	2.51	1.33–4.73	0.0044	1.38	0.64–3.01	0.41
Pathological stage	1-stage	1.35	1.10–1.66	0.0036	1.14	0.88–1.47	0.33
*PD-L1* copy number alterations	Amplification/disomy	19.90	5.73–69.00	< 0.0001	14.20	3.90–52.00	< 0.0001
	Polysomy/disomy	10.50	6.17–18.00	< 0.0001	10.60	5.96–18.80	< 0.0001
Intensity of immune infiltrates	High/low	2.78	1.70–4.56	< 0.0001	3.50	1.95–6.27	< 0.0001
EGFR intensity	High/low	2.77	1.95–3.94	< 0.0001	2.14	1.37–3.36	< 0.001
p-EGFR intensity	High/low	1.78	1.21–2.62	0.0034	1.51	0.92–2.49	0.10
p-Akt intensity	High/low	1.13	0.50–2.57	0.77	0.66	0.24–1.81	0.42
Mutant EGFR expression	Positive/negative	0.46	0.29–0.74	0.0012	0.64	0.35–1.19	0.16
ALK expression	Positive/negative	2.29	0.65–7.99	0.20	3.74	0.95–14.70	0.059

Abbreviations: OR, odds ratio; CI, confidence interval; EGFR, epidermal growth factor receptor; p-EGFR, phospho-EGFR; p-Akt, phospho-Akt; ALK, anaplastic lymphoma kinase.

### Comparison of PD-L1 gene copy number and protein expression between primary tumors and corresponding regional lymph node metastases

Because *PD-L1* copy number gains were significantly associated with PD-L1 expression, we next evaluated and compared the degree of concordance of PD-L1 expression and copy numbers between the primary tumors and the synchronous matched lesions of regional lymph node metastases. Of 173 cases with nodal metastases, 132 lymph node specimens were evaluable for PD-L1 expression. *PD-L1* copy numbers were successfully assessed in 126 lymph node specimens, and both primary and metastatic lesions were successfully interpreted by FISH analyses in 121 cases. There was agreement of PD-L1 expression in 85 (64.4%) paired specimens (kappa = 0.30; 95% CI 0.14–0.45, Table 3). In contrast, a greater level of concordance (105 of 121, 86.8%) in *PD-L1* copy number status was observed between primary tumors and corresponding regional lymph node metastases (weighted kappa = 0.76; 95% CI, 0.65–0.88; Table 4). The agreement and disagreement of PD-L1 expression and copy number status are depicted in the chord diagrams in Supplementary Figure S2.

**Table 3:** Agreement for the PD-L1 expression positivity between primary tumors and metastatic regional lymph nodes

Primary tumors	Lymph node metastases		Total
	Positive	Negative	
Positive	39 (54.9%)	15 (24.6%)	54
Negative	32 (45.1%)	46 (75.4%)	78
Total	71	61	132

Kappa coefficient of agreement = 0.30 (95% confidence interval, 0.14–0.45).

**Table 4:** Agreement for *PD-L1* copy number status between the primary tumors and metastatic regional lymph nodes

Primary tumors	Lymph node metastases			Total
	Disomy	Polysomy	Amplification	
Disomy	87 (94.6%)	7 (28.0%)	0	94
Polysomy	5 (5.4%)	15 (60.0%)	1 (25.0%)	21
Amplification	0	3 (12.0%)	3 (75.0%)	6
Total	92	25	4	121

Weighted kappa coefficient of agreement = 0.76 (95% confidence interval, 0.65–0.88).

### Survival analysis according to PD-L1 expression and *PD-L1* copy number alterations

**Figure 3:**
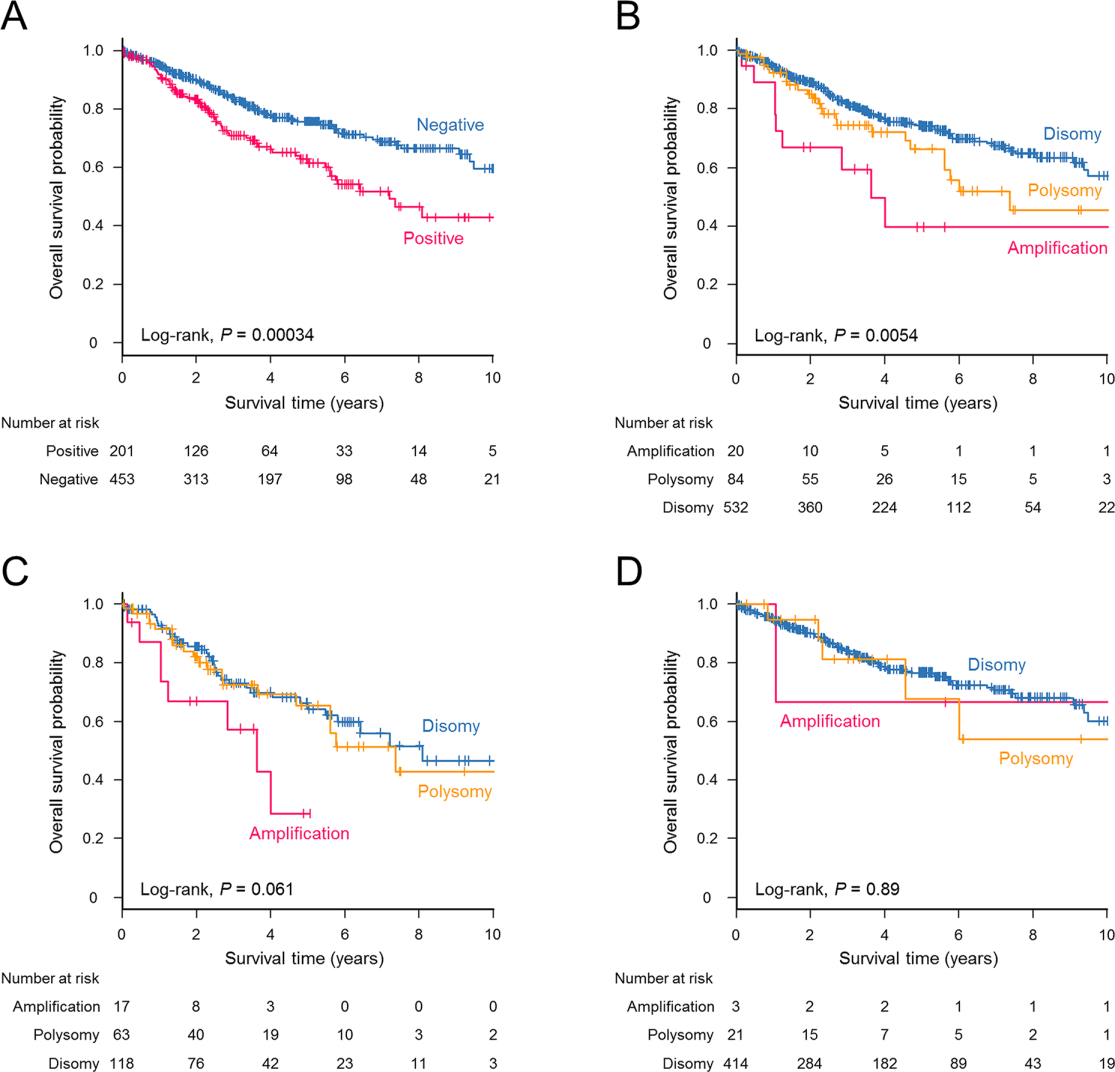
Kaplan–Meier estimates for overall survival (years) stratified according to PD-L1 expression and *PD-L1* copy number status A significant survival difference was observed for NSCLC patients with PD-L1-positive tumors compared with those with PD-L1-negative tumors (log-rank, *P* = 0.00034) (**A**) and for patients with NSCLC according to *PD-L1* copy number alterations (log-rank, *P* = 0.0054) (**B**). (**C**) Patients with *PD-L1* amplification tended to have a poorer survival outcome when the analysis was restricted to PD-L1-positive cases (log-rank, *P* = 0.061). (**D**) No differences in survival were observed among PD-L1-negative patients according to the *PD-L1* copy number status (log-rank, *P* = 0.89).

The estimated median survival time (MST) in the entire cohort was 11.5 years (95% CI, 9.4–not reached (NR)), with a median follow-up duration of 3.1 years. A significant difference in overall survival was observed between PD-L1-positive and -negative patients (log-rank, *P* = 0.00034, Figure 3A). The MST of PD-L1-positive patients was 7.2 years (95% CI, 5.6–NR), whereas that of PD-L1-negative patients was 14.0 years (95% CI, 9.5–NR). Patient survival outcomes also differed significantly according to *PD-L1* copy number alterations (log-rank, *P* = 0.0054, Figure 3B). The MSTs were 3.6 years (95% CI, 1.1–NR) for *PD-L1*-amplified tumors, 7.4 years (95% CI, 5.6–NR) for *PD-L1*-polysomy tumors, and 11.8 years (95% CI, 9.5–NR) for *PD-L1*-disomy tumors. The influence of *PD-L1* copy number increase on overall survival was prominent when restricted to PD-L1 expression-positive patients (Figure 3C). In contrast, no difference in overall survival was observed when only patients with PD-L1-negative tumors were assessed (Figure 3D). Univariate Cox proportional hazards models showed that male sex, smoking history, nonadenocarcinoma histology, disease stage, *PD-L1* amplification, and PD-L1 overexpression had a negative effect on postoperative survival (Table 5). A multivariate analysis indicated that age, nonadenocarcinoma nonsquamous histology, and disease stage were statistically significant predictors of a poor prognosis. *PD-L1* amplification and PD-L1 overexpression did not remain significant in the multivariate analysis (Table 5). Patients with PD-L2 expression-positive tumors did not show a significantly reduced survival compared with PD-L2-negative counterparts (log-rank, *P* = 0.21).

**Table 5:** Results of univariate and multivariate Cox proportional hazards modeling of overall survival by clinicopathological factors, *PD-L1* copy numbers, and PD-L1 expression

Variable	Per unit for HR	Unadjusted HR	95% CI	*P* value	Adjusted HR	95% CI	*P* value
Age (years)	1-year	1.01	1.00–1.03	0.081	1.03	1.01–1.05	0.0043
Sex	Male/female	2.31	1.57–3.40	< 0.0001	1.37	0.77–2.44	0.28
Smoking status	Ever/never	2.91	1.90–4.47	< 0.0001	1.55	0.80–2.99	0.19
Histology	Squamous cell carcinoma/adenocarcinoma	2.24	1.61–3.12	< 0.0001	1.28	0.86–1.89	0.22
	Others/adenocarcinoma	3.70	2.30–5.95	< 0.0001	1.93	1.12–3.32	0.017
Pathological stage	1-stage	2.16	1.82–2.56	< 0.0001	2.06	1.70–2.50	< 0.0001
*PD-L1* copy number alterations	Amplification/disomy	2.63	1.34–5.19	0.0052	1.15	0.54–2.43	0.72
	Polysomy/disomy	1.46	0.95–2.24	0.085	0.83	0.51–1.32	0.43
PD-L1 expression	Positive/negative	1.76	1.29–2.41	< 0.001	1.23	0.86–1.76	0.26

Abbreviations: HR, hazard ratio; CI, confidence interval.

## DISCUSSION

We investigated the association between the *PD-L1* and *PD-L2* copy number gains and expression levels of the corresponding proteins, clinicopathological characteristics, other oncogenic alterations, and survival outcomes in a large cohort of 654 patients with resected NSCLC. For the first time, we noted that a *PD-L1* copy number increase was associated with PD-L1 expression in NSCLC even after adjustments for known associated factors. Importantly, there was a high consistency of *PD-L1* copy numbers in tumor cells between paired primary and metastatic lesions in contrast to a low agreement of PD-L1 expression. Regarding postoperative survival, both amplification of the *PD-L1* gene and PD-L1 overexpression indicated poor prognosis in univariate analyses.

An increase in copy number is a somatic change resulting in the gain of a fraction of DNA regions, which sometimes relates to carcinogenesis and tumor progression. In NSCLC, several important genes are known to be amplified [25] and have been identified as prognostic indicators, mechanisms of drug resistance, or treatment targets. The *PD-L1*, *PD-L2*, and *JAK2* genes are located on chromosome 9p24.1, and their amplifications have been reported in lymphomas [21, 22], an EBV-positive type of gastric cancer [23], and triple-negative breast cancer [24]. An increase in copy numbers can sometimes upregulate the corresponding proteins [26], and the overexpression of PD-L1 may frequently be observed in *PD-L1*-amplified cases in such tumors. In contrast, information on the *PD-L1* and *PD-L2* copy number status was lacking in NSCLC. In the present study, we firstly showed that *PD-L1* and *PD-L2* copy numbers were increased, and *PD-L1* copy number gains were independently associated with PD-L1 expression in NSCLC.

New immunotherapies targeting the PD-1 and PD-L1/L2 pathways to reactivate the suppressed tumor immune system have shown promising results in various cancers. In NSCLC, nivolumab, one of the anti-PD-1 antibodies, has been demonstrated to have an encouraging antitumor effect [11]. Among patients with previously treated advanced squamous cell lung cancer [2] and nonsquamous NSCLC [3], a significantly improved survival benefit and a greater response rate have been observed for nivolumab compared with docetaxel. Similar to nivolumab, promising results have been obtained in trials testing another PD-1 inhibitor, pembrolizumab [13], and a PD-L1 inhibitor, atezolizumab (ClinicalTrials.gov number, NCT01903993), in patients with advanced NSCLC. Significantly, only a subset of patients responded to anti-PD-1/PD-L1 therapy. Biomarkers to predict treatment benefits have been vigorously explored. The nonsynonymous mutation burden, the molecular smoking signature, and the mismatch-repair deficiency of tumors, all of which would result in neoantigen generation, were correlated with the clinical benefits of pembrolizumab [27, 28]. Furthermore, PD-L1 expression on tumor cells and/or on tumor-infiltrating immune cells has been reported to be a potential biomarker of nivolumab [3, 12], pembrolizumab [13], and atezolizumab [14]. However, not all patients with PD-L1-positive tumors responded to these therapies and up to 20% of patients without PD-L1 expression benefit from the therapies [14]. In trials evaluating the efficacy of nivolumab in NSCLC [2, 11], tumor PD-L1 expression levels did not predict a treatment benefit. These conflicting results may be attributed to several factors: differences in demographic and molecular characteristics among the patient cohort, differences in the underlying mechanisms of PD-L1 expression, differences in antibody clones and cutoff values used to determine PD-L1 positivity, differences in targets used to evaluate PD-L1 expression (whether or not PD-L1 expression on tumor-infiltrating immune cells was included), and differences in the condition of tissue fixation and stability of epitopes during immunohistochemistry (IHC) reactions. Moreover, PD-L1 expression is known to be spatially [29] and temporally heterogeneous. In the present study, the agreement of PD-L1 positivity between matched primary and metastatic tumors was poor. We showed that *PD-L1* copy number gains were independent and strong predictors of PD-L1 expression. Moreover, *PD-L1* copy number status had a higher consistency between regional lymph node metastases and primary tumors, indicating that *PD-L1* copy number was sustained less dynamically in cancer cells than PD-L1 expression. In addition, a recent study suggests that *PD-L1* amplification might also increase the level of PD-L1 expression in the adaptive state stimulated by immune cells [30]. These results highlight the significance of copy number analysis of *PD-L1* in NSCLC.

Our findings showed that both PD-L1 expression and *PD-L1* copy number increase were more common among smoking-related NSCLCs. Another study identifies PD-L1 expression more commonly in squamous cell carcinoma than in adenocarcinoma [31]. These results are consistent with findings that tumor-propagating cells in squamous cell carcinoma highly express PD-L1 [20]. Calles et al. [32] report that PD-L1 expression is more frequently observed in smokers and is positively associated with smoking dosage in *KRAS*-mutant NSCLC. By contrast, Azuma et al. [33] show that adenocarcinoma histology is independently associated with PD-L1 expression in a cohort of 164 patients with resected NSCLC. The relationship between these characteristics of lung cancer and PD-L1 expression is controversial, which may be attributed to some methodological issues such as differences in patient cohort, antibody clones, and cutoff values. In addition, several confounding factors in PD-L1 expression complicate their relationship. In our logistic regression analyses, some variables with significant association with PD-L1 expression in univariate analyses lost their significance in the multivariate analysis. As for outcome analyses, PD-L1 overexpression and *PD-L1* amplification lost their significant effect on postoperative survival in the multivariate analysis. The prognostic significance of PD-L1 has been variously reported; it may be associated with better [31, 34, 35] or poorer survival [33, 36]. In the present study, only age, nonadenocarcinoma nonsquamous histology, and disease stage were significantly prognostic when adjusted for confounders. These conflicting results can be explained by differences in sample size, disease stage, race, underlying etiology of PD-L1 expression, methods to assess PD-L1 expression, and threshold of PD-L1 positivity. Further prospective studies using a large sample size and a standardized method for the evaluation of PD-L1 expression levels and *PD-L1* copy number status are needed to clarify the definitive prognostic significance of PD-L1.

In contrast to PD-L1 that is constitutively expressed by antigen-presenting cells, nonhematopoietic cells, and other many organs, PD-L2 expression which is less prevalent than PD-L1 in tumors is relatively restricted to macrophages, dendritic cells, and fibroblasts [37]. Compared with PD-L1, there is little information on the mechanisms regulating PD-L2 expression in tumors. In the present study, PD-L2 expression had no association with copy number gains of the *PD-L2* gene. This could possibly be explained by the low affinity of the antibody used for IHC, resulting in lower PD-L2 positivity than PD-L1 and through gene silencing by epigenetic modifications such as DNA methylation and histone deacetylation. As a result, despite the status of copy numbers of *PD-L1* and *PD-L2* being closely matched, the relationship between PD-L1 and PD-L2 expression was discordant in more than half of the PD-L2-positive specimens. The *JAK2* copy number was also co-altered with the *PD-L1* copy number. The copy number-dependent JAK-STAT activity is known to induce further PD-L1 expression in lymphoma [21]. In addition, PD-L1 expression of the *PD-L1*-amplified lung cancer cell line, HCC4006, is shown to be reduced by JAK2-specific and STAT3-specific inhibitors [30]. In the present study, however, copy number gains of *JAK2* were not positively associated with expression of p-STAT3 in tumor cells. Further studies are needed *in vivo* to clarify whether JAK2 is activated in a copy number-dependent manner in NSCLC.

This study had several limitations. First, the threshold used to define PD-L1 positivity has not been validated. The threshold may differ according to antibodies used, the size of the specimens, disease stage, and race. The positive rate of PD-L1 protein expression in this study was similar to those in previous reports [3, 34]. Clone E1L3N that was used in the present study has been well validated for formalin-fixed and paraffin-embedded (FFPE) tissue and demonstrated to be the most sensitive for membranous PD-L1 in IHC of NSCLC specimens with little cytoplasmic staining among several other clones [38]. Ultimately, the threshold should be determined as the clone-specific value that can most accurately discriminate between responses to anti-PD-1/PD-L1 therapy. Second, a confounding bias is inevitable because of the retrospective nature of this study. To minimize the influence, however, we recruited a large number of patients and used a solid outcome endpoint: overall survival. Finally, not all *EGFR* mutations and *ALK* rearrangements were analyzed in a standard manner, such as PCR-based assays for *EGFR* and FISH or RT-PCR assays for *ALK*. We evaluated both gene alterations using IHC methods, which are inferior to standard methods. The sensitivity of IHC is known to be low, especially for the detection of non-15-base pair deletions (E746–A750) in exon 19 for *EGFR* [39]. However, even when only 267 patients whose tumor *EGFR* mutation status had been molecularly determined by standard methods were examined, clinicopathological features based on PD-L1 expression and *PD-L1* copy number were similar to results from the entire cohort (Supplementary Table S4). Contrary to *EGFR* mutations, the evaluation of ALK positivity using IHC has been shown to be sufficiently capable of detecting *ALK* rearrangements. When FISH is used as the gold-standard reference, the sensitivity and specificity of ALK-IHC have been reported as 90% and 97.8%, respectively [40]; the present study used the same definition.

In conclusion, a *PD-L1* copy number increase was demonstrated to be a strong and independent factor associated with PD-L1 expression in a large number of surgically treated patients with NSCLC. Because PD-L1 expression has yet to be confirmed as a predictor of response to anti-PD-1/PD-L1 treatment and PD-L1 expression status varies both spatially and temporally, an exploration of alternative or complementary biomarkers is important for the rational use of this promising treatment. Our results showed that *PD-L1* copy number gains, as assessed by a FISH assay, might be used as alternative biomarkers with reliable accuracy and reproducibility using archival samples. Further studies are required to determine whether *PD-L1* copy numbers are correlated with a clinical benefit of anti-PD-1/PD-L1 therapy.

## MATERIALS AND METHODS

### Tumor collection and tissue microarray construction

This study included two independent cohorts of surgically treated patients with NSCLC: 427 patients who underwent surgical resection between January 1990 and December 2013 at Hamamatsu University Hospital (Japan) and 227 patients who underwent surgery between January 2006 and April 2014 at Seirei Mikatahara General Hospital (Japan). All of the patients were Japanese and provided written informed consent for the use of resected specimens for medical research. The study design was approved by the Institutional Review Boards of Hamamatsu University School of Medicine and Seirei Mikatahara General Hospital. The investigation was conducted in accordance with ethical standards and according to the Declaration of Helsinki as well as national and international guidelines. Clinical and pathological data including outcomes were retrospectively obtained from a review of the patients’ medical records. Three board-certified pathologists (KS and HS at Hamamatsu University Hospital and HO at Seirei Mikatahara General Hospital) classified the lung cancers according to the WHO classification (7th edition). Tissue microarrays (TMAs) in which individual cores had a diameter of 2 or 3 mm were isolated from representative lung cancer tissue, and all the TMA cores were validated to contain a sufficient number of tumor cells by reviewing hematoxylin and eosin (HE)-stained sections, as reported previously [25, 41]. To obtain TMAs from metastatic regional lymph nodes, we selected specimens located at lymph node stations furthest from the primary tumors, if multiple stations were metastatic.

### FISH and copy number assessment

**Figure 4:**
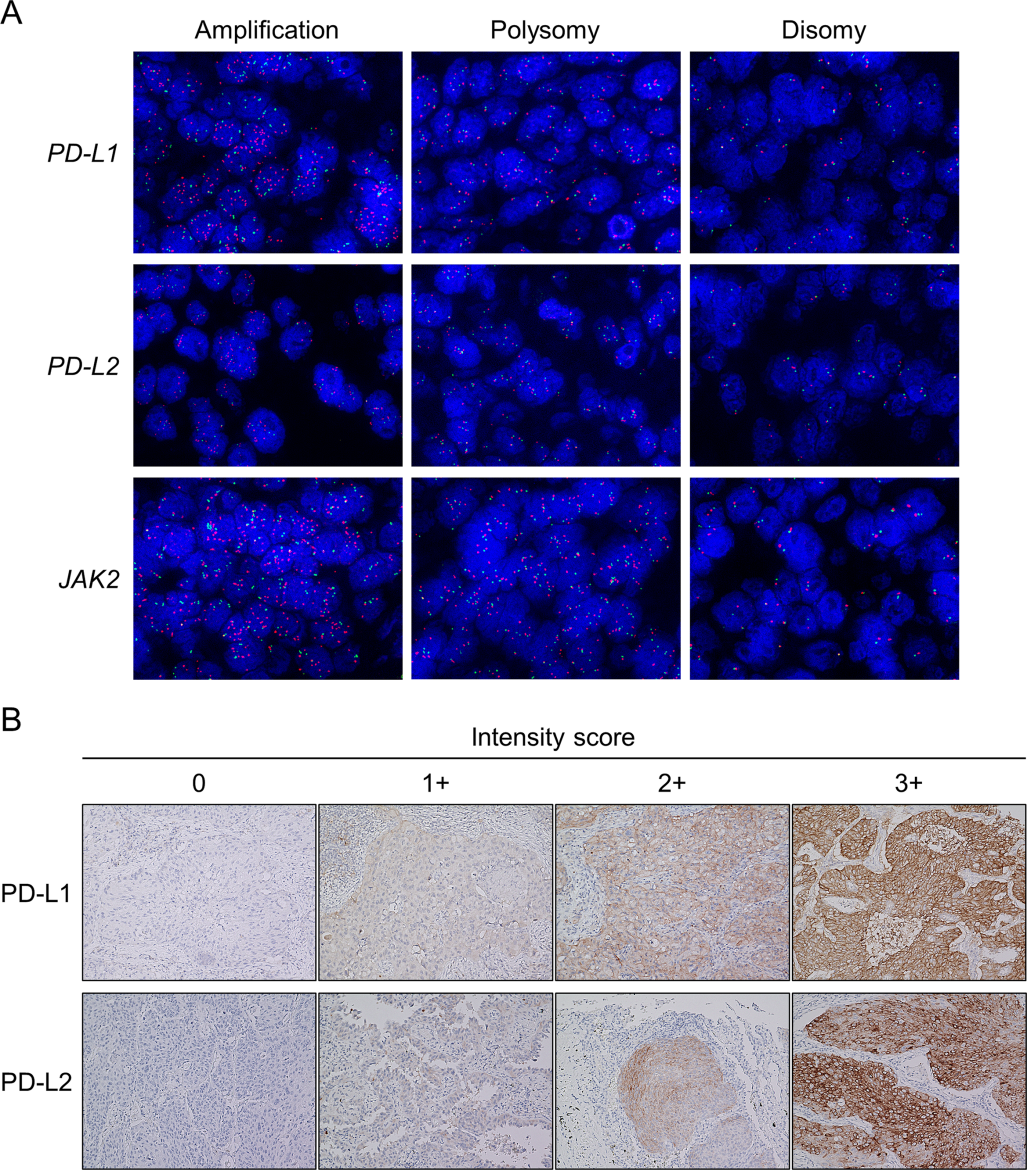
Representative images of fluorescence *in situ* hybridization (FISH) for the *PD-L1, PD-L2*, and *JAK2* genes and representative images of the immunohistochemistry (IHC) analysis for PD-L1 and PD-L2 In the FISH panel (**A**), the upper, middle, and lower images show the *PD-L1*, *PD-L2*, and *JAK2* copy number variants in non-small-cell lung cancer, respectively. Images show amplification (left), polysomy (middle), and disomy (right; original magnification × 100). The *PD-L1*, *PD-L2*, and *JAK2* genes are shown in red, and the CEP9 is represented in green. In the IHC panel (**B**), each tumor membranous intensity of 0 (absent), 1 (weak), 2 (moderate), and 3 (strong) for PD-L1 staining and each tumor membranous and/or cytoplasmic intensity of 0 (absent), 1 (weak), 2 (moderate), and 3 (strong) for PD-L2 staining are given (original magnification × 20).

FISH analyses were performed using TMA sections according to the manufacturers’ instructions, as previously described [25, 42, 43]. Spectrum Orange-labeled bacterial artificial chromosome (BAC) clones, RP11-599H20 (9p24.1, *PD-L1*), RP11-635N21 (9p24.1, *PD-L2*), and RP11-982A21 (9p24.1, *JAK2*; Advanced GenoTechs Co., Tsukuba, Japan), were used as locus-specific FISH probes. The spectrum Green-labeled control probe for the near-centromere locus on chromosome 9 (RP11-113O24) (Advanced GenoTechs Co.) was used to enumerate chromosome 9. For nuclear staining, 4′,6-diamidino-2-phenylindole (Vector Laboratories, Burlingame, CA) was used. The probes used in this study had been validated by hybridization to the chromosomal metaphase spread of normal lymphocytes to verify chromosomal numbers and loci. Interpretation of FISH signals was performed without reference to specimen identification. The cores with signals with insufficient intensity for interpretation were excluded from the analyses. After screening the entire area of individual cores, the probe signals for a monolayer of at least 50 tumor cell nuclei were counted at ×100 magnification in at least three representative images per case. The mean values of the target signals (*PD-L1*, *PD-L2*, and *JAK2*) and the CEP9 signals, as well as the mean target BAC signal/CEP9 signal ratios, were calculated. Copy number amplification was defined according to the average target BAC signal/CEP9 signal ratio of ≥ 2.0 (Figure 4A) [25]. Polysomy was defined based on the criteria that a mean copy number of the target signals was ≥ 3.0, and the ratio to CEP9 signals was < 2.0 (Figure 4A). Among cases of polysomy, specimens that had target signals of ≥ 4.0 were classified as “high polysomy,” and the others were classified as “low polysomy.” All other tumors were considered to exhibit disomy (Figure 4A), of which specimens with an average target signal of ≥ 2.5, but < 3.0 copies were subclassified as “borderline polysomy.” FISH interpretation was performed using a Z-stacked two-dimensional picture with a fluorescence microscope (BZ-9000; KEYENCE, Osaka, Japan); image contrast was adjusted for the entire area.

### IHC staining and evaluation

TMA blocks composed of 50 cores per slide were analyzed for PD-L1 and PD-L2 by IHC. Furthermore, we semiquantified the infiltrated immune cells and evaluated expression levels of other proteins such as total EGFR, p-EGFR, phospho-Akt (p-Akt), mutant EGFR with the exon 19 deletion or the L858R point mutation, and ALK using IHC, all of which have been previously reported to be associated with PD-L1 overexpression in NSCLC tumor cells [17–20, 44, 45]. IHC analyses were performed as described in our previous report [46]. Briefly, FFPE sections were deparaffinized and rehydrated. After antigen retrieval was performed, endogenous peroxidase was quenched with hydrogen peroxide before reacting with primary antibodies against PD-L1 (dilution 1:100, clone E1L3N; Cell Signaling Technology (CST), Danvers, MA, USA), PD-L2 (dilution 1:50, clone D7U8C; CST), p-EGFR (Tyr1068, dilution 1:200, clone D7A5; CST), p-Akt (Ser473, dilution 1:50, clone D9E; CST), p-STAT3 (Tyr705, dilution 1:100, clone D3A7; CST), and mutant EGFR with the exon 19 deletion (E746-A750; dilution 1:100, clone 6B6; CST) or the L858R point mutation (dilution 1:100, clone 43B2; CST) for 30 min at room temperature. For the total EGFR staining, treatment with proteinase K solution for 5 min at room temperature followed by peroxidase blocking was performed before reaction with the primary antibody against EGFR (dilution 1:50, clone 31G7; Zymed Laboratories Inc., South San Francisco, CA, USA). For ALK staining, the intercalated antibody-enhanced polymer method [47] was used (anti-ALK antibody: dilution 1:50, clone 5A4; Abcam, Cambridge, UK). After two washes, antigen–antibody complexes were visualized using Histofine Simple Stain Max PO kit (Nichirei, Tokyo, Japan) and 3, 3′-diaminobenzidine tetrahydrochloride. After two additional washes, nuclear counterstaining was performed with hematoxylin. YI and KY independently evaluated protein expression levels in a blinded manner as during FISH interpretation, and a consensus was obtained in cases of discrepancy. For PD-L1, the semiquantitative H-score was calculated by multiplying the membranous intensity score (0, absent; 1, weak; 2, moderate; 3, strong, Figure 4B) by the percentage of stained cells (from 0% to 100%). Thus, the H-score could range from 0 to 300. Specimens with an H-score ≥ 5 were defined as PD-L1 positive, because a previous study had established this cutoff value using the same antibody E1L3N [45], and this cutoff value was almost equivalent to the cutoff of a 5% expression threshold used in other clinical trials and studies [12, 48]. Different from PD-L1, the H-score of PD-L2 was calculated by multiplying the membranous and/or cytoplasmic intensity score (0, absent; 1, weak; 2, moderate; 3, strong, Figure 4B) by the percentage of stained cells, because PD-L2 expression was both membranous and cytoplasmic as reported in previous studies [32, 49]. Similar to PD-L1, specimens with an H-score ≥ 5 were classified as PD-L2 positive. The intensity of immune infiltrates was assessed on HE-stained sections and was scored from 0 to 3 according to the definitions used in previous reports [48, 50] with modifications: 0, no immune infiltrates; 1, mild stromal immune infiltrates (occupying < 50% of the stromal surface area); 2, moderate stromal immune infiltrates (occupying ≥ 50% of the surface area); and 3, strong immune infiltrates obscuring the tumor. Scores of 0 and 1 were considered to represent “low” immune infiltrates, and scores of 2 and 3 were considered “high” immune infiltrates. The IHC of EGFR was graded as follows [51]: 0, no staining or membrane staining in < 10% of tumor cells; 1, partial, faint membrane staining in ≥ 10% of tumor cells; 2, weak-to-moderate staining in ≥ 10% of tumor cells; and 3, strong complete membrane staining in ≥ 10% of tumor cells. Scores of 0 and 1 were considered to represent “low” EGFR expression, and scores of 2 and 3 were considered to represent “high” EGFR expression. Membranous p-EGFR immunostaining was assigned a semiquantitative score: 0, < 5% positive cells;1, 5%–19% positive cells; 2, 20%–50% positive cells; and 3, > 50% positive cells [51]. Specimens with a score of 1–3 were considered “high,” and those with a score of 0 were considered “low.” For p-Akt, each specimen was graded as 0 if no staining was observed, as 1 if more than 5% of the tumor cells showed cytoplasmic or weak nuclear staining, and as 2 if more than 5% of the cells exhibited strong nuclear staining [52]. Specimens with a score of 2 were considered to represent “high” p-Akt. Staining for p-STAT3 was considered “high” if > 25% of tumor nuclei exhibited moderate or strong intensity of staining [53]. For the assessment of mutant EGFR IHC, the specimens were graded as follows: 0, no staining or faint staining in < 10% of tumor cells; 1, faint staining in > 10% of cells; 2, moderate staining; and 3, strong staining [39, 54]. Tumors with grades 1–3 were considered “positive” and those with grade 0 were considered “negative” [54]. The ALK staining intensity was interpreted according to the following criteria: 0, no staining; 1, faint cytoplasmic staining in > 10% of tumor cells; 2, moderate cytoplasmic staining; and 3, strong cytoplasmic staining [40]. Tumors with grades of 2 or 3 were defined as ALK “positive” [40].

### Assessment of *EGFR* mutation status

Of the 654 recruited patients, *EGFR* mutation analyses were performed as part of clinical practice in commercial clinical laboratories (SRL in Tokyo (cycleave method), LSI Medience in Tokyo (peptide nucleic acid-locked nucleic acid PCR clamp method or the cobas EGFR assay), or BML, Inc. in Tokyo (PCR invader method)) for only 267 patients. Therefore, we assessed the *EGFR* mutation status of all the specimens using IHC with two antibodies specific for the E746-A750 deletion in exon 19 and for the exon 21 L858R mutation. In the present study, the sensitivity, specificity, and positive and negative predictive values for mutant EGFR specific IHC, compared with *EGFR* mutation status (exon 19 deletions or L858R mutation in exon 21), as determined using the above-mentioned PCR-based methods, were 77.3%, 94.3%, 84.1%, and 91.4%, respectively.

### Statistical analysis

The Fisher's exact test for categorical variables and the Mann–Whitney *U* test or the Kruskal–Wallis test for continuous variables were used. *P* values in multiple comparisons were adjusted using the method of Holm. The extent of agreement was assessed using Cohen's weighted kappa statistics or kappa statistics. Chord diagrams were used to visualize similarities and differences of agreement. Logistic regression was performed to analyze the association between PD-L1 expression and the *PD-L1* copy number gains with adjustment for confounders. Overall survival was defined as the time between the date of operation and the date of death or last contact. The survival curves were estimated using the Kaplan–Meier method, and the log-rank test was used to analyze differences in survival time. Cox proportional hazard regression analyses were fitted to determine the impact of risk factors on overall survival with adjustment for other potential confounding factors. The proportional hazard assumption was assessed based on Schoenfeld residuals. Statistical tests were two sided, and *P* values < 0.05 were considered to be statistically significant. All data analyses were performed using R (R Foundation for Statistical Computing, Vienna, Austria, version 3.2.0) and additional packages from the comprehensive R archive network (CRAN) library.

## SUPPLEMENTARY MATERIALS FIGURES AND TABLES






