# Supplementary material for: Clinical significance of PD-L1 and PD-L2 copy number gains in non-small-cell lung cancer
Source: Oncotarget. 2016 Apr 1;7(22):32113–28. doi: 10.18632/oncotarget.8528 (PMC5078001; doi:10.18632/oncotarget.8528)
Supplement: Supplementary file 1 [file oncotarget-07-32113-s001.pdf]

Clinical significance of *PD-L1* and *PD-L2* copy number gains in non-small-cell lung cancer

Supplementary Materials

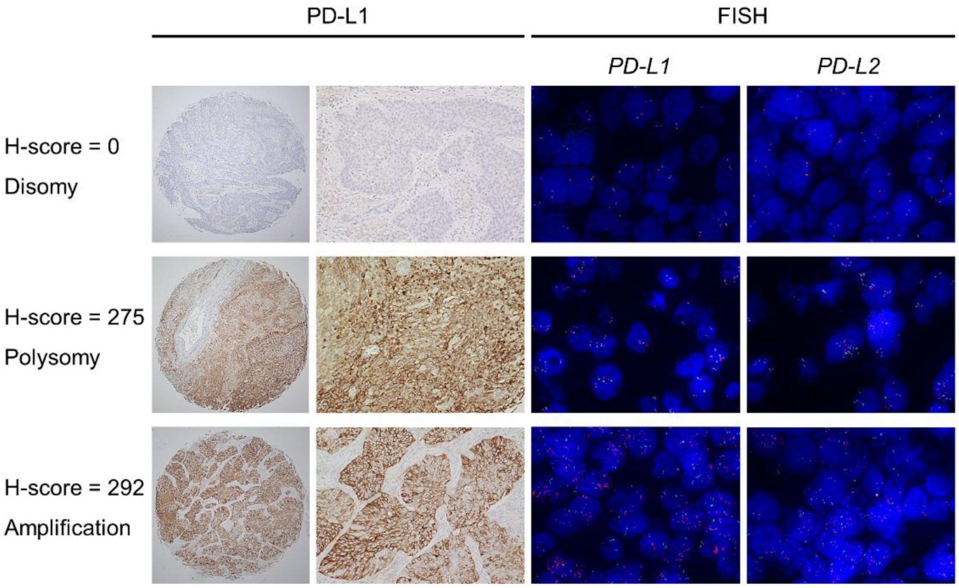

Supplementary Figure S1: Representative images of immunohistochemistry analysis of PD-L1 and fluorescence *in situ* hybridization (FISH) analyses of *PD-L1* and *PD-L2*. The upper case shows no PD-L1 expression and normal copy numbers of the *PD-L1* and *PD-L2* genes. The middle case shows strong PD-L1 expression (H-score = 275) and polysomy of both the *PD-L1* and *PD-L2* genes. The lower case shows images of strong PD-L1 expression (H-score = 292) and amplified-*PD-L1* and -*PD-L2*. The left-side and right-side immunohistochemistry photomicrographs are the original magnification of ×4 and ×20, respectively. The FISH images are the original magnification of × 100.

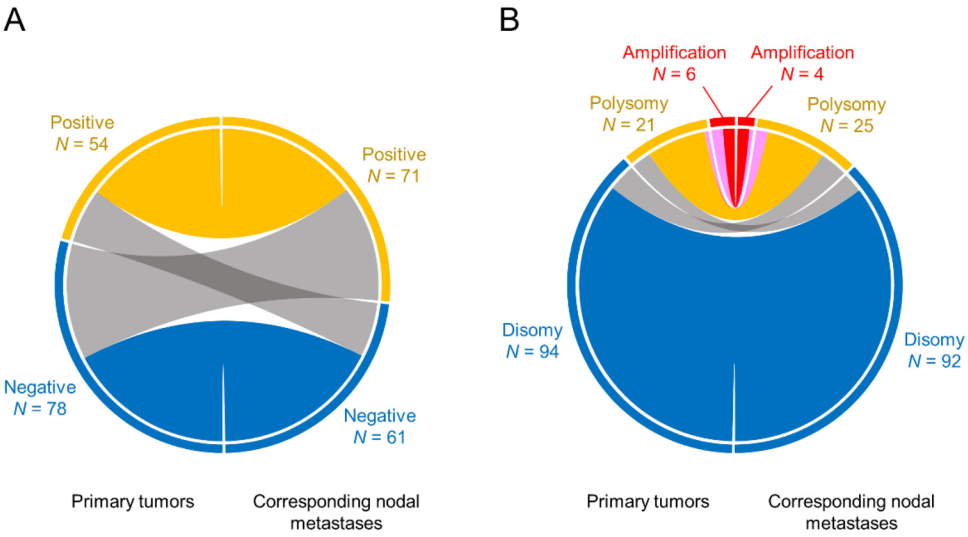

Supplementary Figure S2: Chord diagrams summarizing the agreement and disagreement of PD-L1 statuses between primary tumors and matched metastatic regional lymph nodes. Primary tumors are depicted in the left side, and lymph node metastases are depicted in the right side of each chord diagram. The agreement of PD-L1 expression positivity is shown on the left side of the chord diagram (**A**), and that of *PD-L1* copy number status is shown on the right side (**B**). The blue, yellow, and red colored areas represent concordance, and gray and pink colored areas represent discordance.

Supplementary Table S1: Clinicopathological characteristics of patients with non-small-cell lung cancer related to PD-L2 expression and *PD-L2* copy number status. See Supplementary_Table_S1

Supplementary Table S2: Agreement for copy number alterations divided into five groups between the *PD-L1* and *PD-L2* genes

	PD-L2 copy number					
PD-L1 copy number	Disomy	Borderline polysomy	Low polysomy	High polysomy	Amplification	Total
Disomy	502 (96.7%)	4 (19.0%)	3 (7.5%)	0	0	509
Borderline polysomy	9 (1.7%)	9 (42.9%)	0	0	0	18
Low polysomy	6 (1.2%)	7 (33.3%)	22 (55.0%)	3 (8.1%)	1 (9.1%)	39
High polysomy	1 (0.2%)	1 (4.8%)	13 (32.5%)	28 (75.7%)	0	43
Amplification	1 (0.2%)	0	2 (5.0%)	6 (16.2%)	10 (90.9%)	19
Total	519	21	40	37	11	628

Weighted kappa coefficient of agreement = 0.91 (95% confidence interval, 0.87–0.94).

Supplementary Table S3: Agreement for copy number alterations divided into five groups between the *PD-L1* and *JAK2* genes

	JAK2 copy number					
PD-L1 copy number	Disomy	Borderline polysomy	Low polysomy	High polysomy	Amplification	Total
Disomy	474 (95.8%)	23 (62.2%)	6 (14.0%)	2 (5.9%)	0	505
Borderline polysomy	11 (2.2%)	7 (18.9%)	0	0	0	18
Low polysomy	8 (1.6%)	5 (13.5%)	21 (48.8%)	5 (14.7%)	1 (6.25%)	40
High polysomy	2 (0.4%)	2 (5.4%)	14 (32.6%)	24 (70.6%)	1 (6.25%)	43
Amplification	0	0	2 (4.6%)	3 (8.8%)	14 (87.5%)	19
Total	495	37	43	34	16	625

Weighted kappa coefficient of agreement = 0.87 (95% confidence interval, 0.83–0.91).

Supplementary Table S4: Clinicopathological characteristics of patients with non-small-cell lung cancer tested for *EGFR* mutations related to PD-L1 expression and *PD-L1* copy number status. See Supplementary_Table_S4
